# Combining Cellulose and Cyclodextrins: Fascinating Designs for Materials and Pharmaceutics

**DOI:** 10.3389/fchem.2018.00271

**Published:** 2018-07-05

**Authors:** Tânia F. Cova, Dina Murtinho, Alberto A. C. C. Pais, Artur J. M. Valente

**Affiliations:** Coimbra Cemistry Centre, CQC, Department of Chemistry, Faculty of Sciences and Technology, University of Coimbra, Coimbra, Portugal

**Keywords:** cellulose derivatives, cyclodextrins, host-guest interactions, supramolecular chemistry, materials science, drug delivery

## Abstract

Cellulose and cyclodextrins possess unique properties that can be tailored, combined, and used in a considerable number of applications, including textiles, coatings, sensors, and drug delivery systems. Successfully structuring and applying cellulose and cyclodextrins conjugates requires a deep understanding of the relation between structural, and soft matter behavior, materials, energy, and function. This review focuses on the key advances in developing materials based on these conjugates. Relevant aspects regarding structural variations, methods of synthesis, processing and functionalization, and corresponding supramolecular properties are presented. The use of cellulose/cyclodextrin conjugates as intelligent platforms for applications in materials science and pharmaceutical technology is also outlined, focusing on drug delivery, textiles, and sensors.

## Introduction

Cyclodextrins (CDs) and cellulose are fascinating molecules with synergistic properties that have been critical for preparing new derivatives directed at several innovative applications, ranging from textiles (El Ghoul et al., 2016; Mihailiasa et al., 2016) coatings (Huang et al., 2013; Shah et al., 2015; Mihailiasa et al., 2016; Umoren and Eduok, 2016; Jimenez et al., 2017), sensors (Qiu and Hu, 2013) and paper making to pharmaceutical technology (Siepmann and Peppas, 2001; Hadjichristidis et al., 2003; Zhang et al., 2012; Shokri and Adibkia, 2013; Yang and Yang, 2013; Mogosanu and Grumezescu, 2015; Thakur, 2015; Lalatsa and Barbu, 2016; Jawaid and Mohammad, 2017; Nishio et al., 2017; Yadav et al., 2018). Cellulose has been considered a suitable matrix for preparing polymers containing CDs. These combine the favorable properties of cellulose adsorption and wettability with the capacity of CDs to form inclusion complexes with a wide range of guest molecules, including hydrocarbon amphiphiles and drugs (Guo et al., 2013; Medronho et al., 2013; Cova et al., 2017; Zhu et al., 2017).

In general, in this type of structures, CDs can be covalently attached to the surface of cellulose so that the cavity of the former is accessible to the guest molecules. These materials are also used for separation and isolation purposes, being employed as sorbents for different compounds, in solution and in gas phase (Zhang et al., 2012; Fanali, 2017).

Several research groups have opened the way for future progress through innovative applications of cellulose and cyclodextrins directed toward physical and organic chemistry, materials design, as well as pharmaceutical technology. In light of the importance of understanding the relationship between structure and properties, selective modifications have been performed, providing new insights on potential applications, since the type of functionalization and substituents affect the resulting physical properties. A deep understanding of these properties is crucial for designing intelligent systems for drug delivery, as an example of application.

This review describes, in a critical and comprehensive way, relevant contributions carried out recently and involving these two molecules. An exhaustive account of the wide range of works published in the early years of cellulose and cyclodextrin derivatives falls beyond the scope of this review. The reader is referred to references (Siepmann and Peppas, 2001; Hadjichristidis et al., 2003; Zhang et al., 2012; Qiu and Hu, 2013; Shokri and Adibkia, 2013; Yang and Yang, 2013; Liu et al., 2015a; Mogosanu and Grumezescu, 2015; Thakur, 2015; Krukiewicz and Zak, 2016; Lalatsa and Barbu, 2016; Jawaid and Mohammad, 2017; Nishio et al., 2017; Yadav et al., 2018) for a full description of these efforts.

Literature regarding cellulose, cellulose derivatives and **CDs** is quite-extensive and also highly specialized. Data on the applications of **CD**-cellulose-based systems, published in the period from the beginning of 2000 to March, 2018, are summarized in the following sections. However, when considered fundamental, some works published before this period are also mentioned. The diversity of publication titles in which these type of systems are reported (more than 100 journals according to the Web of Science database) makes it difficult for researchers to encompass the latest advances in the field. The relevant advances on the methods of preparation, characterization and application of functional structures involving cyclodextrin and cellulose derivatives, have been described in an uncorrelated manner in the current state of the art, thus being summarized in references (Du et al., 2012; Qiu and Hu, 2013; Yang and Yang, 2013; Liu et al., 2015a; Mogosanu and Grumezescu, 2015; Nafee et al., 2015; Higashi et al., 2016; Lalatsa and Barbu, 2016). An analysis of the number of articles (including books and reviews), on the topics of cellulose derivatives and CD-cellulose-based systems, reported in the Web of Science database(Reuters)^1^ indicates that ca. 13220 and 291 works were published, respectively. In the former set ca. 62% of the whole publications are assigned to Chemistry. The remaining publications are distributed among Biochemistry and Molecular Biology (46%), Polymer Science (41%), Materials Science (34%), Pharmacology and Pharmacy (33%), and Engineering (25%), with additional areas in which the topic is less frequent. A relevant outcome is the number of works focused on the combination of cellulose derivatives and supramolecular macrocycles such as CDs which are protected by ca. 75 patents (data retrieved from Web of Science and Espacenet, EPO, databases).

## Why using cellulose and cyclodextrin derivatives?

**Figure 1:**
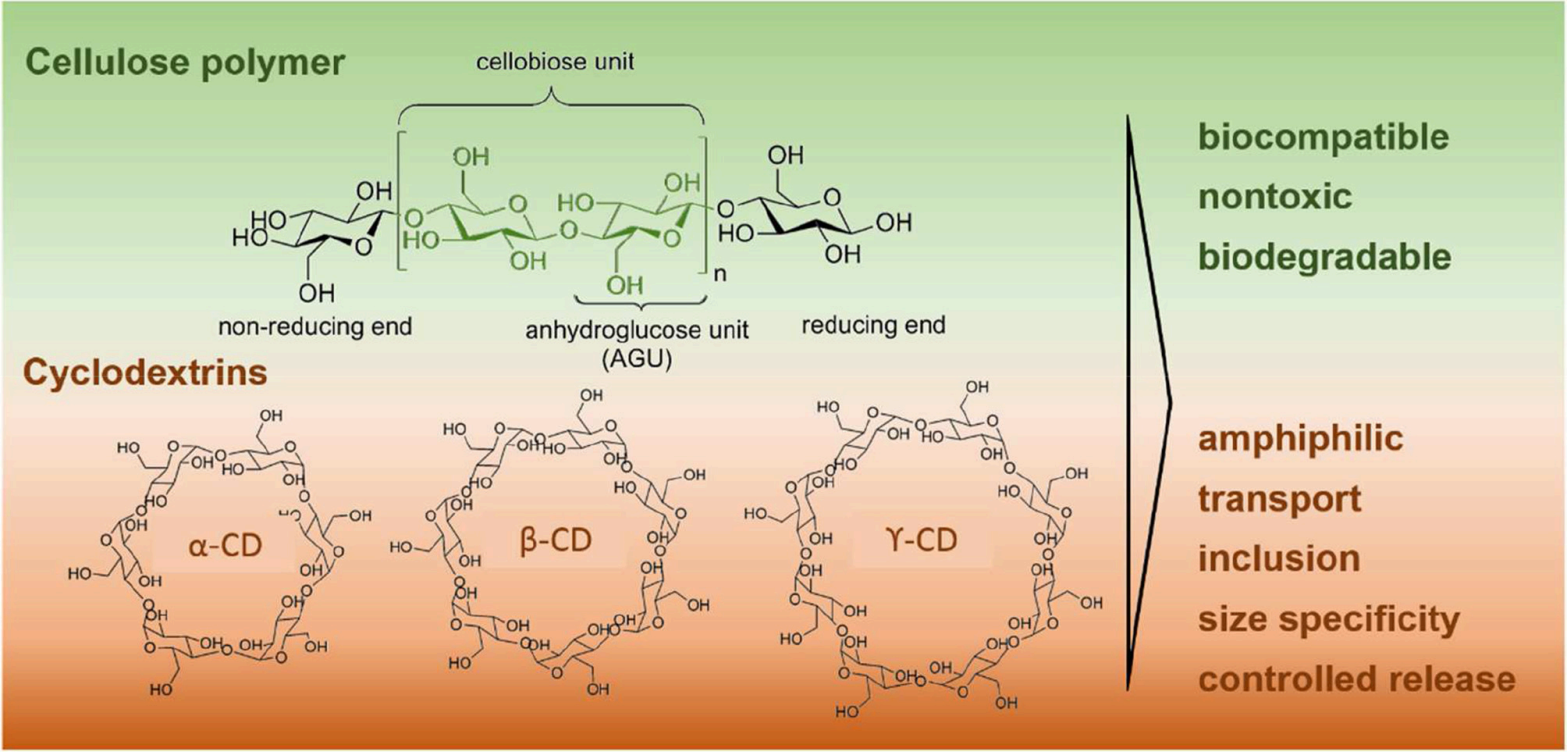
(**Top**) The cellulose polymer chain structure. (**Bottom**) Representation of the molecular structure of the alpha- (α-CD), beta- (β-CD) and gamma-cyclodextrins (γ-CD).

Cellulose is a bipolymer (Figure 1) used as building material in a variety of applications (Mogosanu and Grumezescu, 2015; Lalatsa and Barbu, 2016). Cellulose derivatives belong to an important group of sustainable materials which have been attracting increasing attention of researchers and manufacturers alike.

The hydroxyl groups in the cellulose backbone (Figure 1) can react with different molecules through esterification, etherification and oxidation, affording derivatives with excellent properties (Medronho et al., 2013; Qiu and Hu, 2013; Zhang et al., 2013; Liu et al., 2015a; Mogosanu and Grumezescu, 2015; Thakur, 2015; Lalatsa and Barbu, 2016; Ndong Ntoutoume et al., 2016; Umoren and Eduok, 2016; Jawaid and Mohammad, 2017; Jimenez et al., 2017; Sun et al., 2017b).

Macromolecules such as CD immobilized polysaccharides (Yang and Yang, 2013), obtained from covalent and non-covalent binding strategies, encompass important features, including an excellent biocompatibility (Du et al., 2012; Boztas et al., 2013; Liu et al., 2015a), nontoxicity (Krukiewicz and Zak, 2016), thermal and chemical stability (Wintgens et al., 2012; Iohara et al., 2017), high hydrophilicity (Lin and Dufresne, 2013; Sá-Barreto et al., 2013; Zhang et al., 2013), and biodegradability of the polysaccharides, as well as the unique amphiphilic and transport properties (Guo et al., 2013; Yang and Yang, 2013), and the ability for inclusion (Zhang et al., 2012; Guo et al., 2013; Liu et al., 2014; Nardello-Rataj and Leclercq, 2014; Higashi et al., 2016; Muankaew et al., 2017), size specificity (Higashi et al., 2016; Cova et al., 2018) and controlled release of CDs (Blanchemain et al., 2012; Lavoine et al., 2014b; Han et al., 2017). These latter, presented in Figure 1, are cyclic oligosaccharides with hydrophobic cavities and hydrophilic outer surfaces, which have been recognized as useful matrices. For instance, inclusion complexes based on CDs have been successfully used to improve the water solubility of drugs, aiming at better therapeutic efficacy. Such host-guest systems are capable of modifying the properties of the guest molecules, in particular those related with chemical reactivity and photochemical/thermal degradation, altering the chemical activity of the delivered drugs, and releasing them in a controlled manner, upon competitive binding or external stimulus (e.g., temperature and pH). Their potential for aggregation have also lead to the manufacture of nanoparticles (Higashi et al., 2016; Lalatsa and Barbu, 2016; Mihailiasa et al., 2016; Ndong Ntoutoume et al., 2016) and films (Thomsen et al., 2017), paving the way to applications involving separation processes (Fanali, 2017), drug delivery (Boztas et al., 2013; Namgung et al., 2014; Brackman et al., 2016; Rivera-Delgado and Von Recum, 2017), tissue engineering (Yang and Yang, 2013), among others.

### Cyclodextrins

Cyclodextrins are cyclic oligosaccharides that belong to a group of structurally related natural materials, formed during bacterial digestion of cellulose. These consist of (α-1,4)-linked α-D-glucopyranose units and possess a hydrophobic cavity and a hydrophilic outer surface. Typical CDs are constituted by six (α-CD), seven (β-CD), and eight (γ-CD) glucopyranoside units. Recently (Zhu et al., 2017), these macromolecules, and especially α-CD, have also been recognized as potential kinetic surrogates for cellulose, comparing the glycosidic bond cleavage of α-CD exhibited at low and high temperature kinetic regimes with cellulose hydrolysis.

The first reference to what would later prove to be **CDs** was published in 1891 by Villiers (1891). From the digestion of starch by *Bacillus amylobacter*, Villiers obtained a crystalline substance at which he named “*cellulosine*” due to its similarity toward cellulose, with respect to the acid resistance and the absence of reducing properties. The author has also found two different distinct forms of *cellulosine*, which could be related with the occurrence of α- and β-**CD** forms. One decade later, two different crystalline products resulting from the digestion of potato starch by *Bacillus macerans* were reported by Schardinger (1904, 1905); these compounds were further characterized by using triiodide solutions and were known as by Schardinger dextrin. The ability of these compounds to complex with organic compounds was firstly reported by Prignsheim (Pringsheim and Abbe, 1932) and, in the following decades, it was found that the Schradinger dextrins are a family of natural oligosaccharides formed by 6, 7, or 8 α-(1,4) linked glucopyranose units, denoted as α-, β-, or γ-**CD**, respectively (Freudenberg and Rapp, 1936; Freudenberg et al., 1936, 1938; French, 1957). However, the work carried out by Szejtli (1998) has been considered a landmark for the large scale use of CDs, in particular for their ability to form inclusion (host-guest) supramolecular complexes with a large variety of molecular guests. Such interest is a direct consequence of the structure of CDs. Each of the chiral glucose units in the CD molecules is in the rigid ^4^C_1_-chair conformation, giving the macrocycle the shape of a truncated cone with internal cavity diameter ranging from 6 to 10 Å (Table 1) (Messner et al., 2010). The CD cavity is lined by the hydrogen atoms and the glycosidic oxygen bridges. The nonbonding electron pairs of the glycosidic oxygen bridges are directed toward the cavity interior, conferring some Lewis base character. Simultaneously, the hydroxyl groups are positioned outside at the edges of the truncated cone (Saenger et al., 1998). The secondary hydroxyls are responsible for intramolecular H-bonds between adjacent glucopyranose unities which, as consequence, cannot rotate. The intramolecular H-bonding of secondary hydroxyls, resulting in relatively rigid chains, may contribute for the low solubility of β-CD (Table 1), when compared to α- and γ-CD. In fact, the formed secondary H-bond ring is incomplete in the α-CD (only 4 H-bonds out of 6 are formed); on the other hand the γ-CD is non-planar being both the more flexible and the more soluble of all three natural CDs (Nardello-Rataj and Leclercq, 2014).

**Table 1:** Some physical properties of α-, β- and γ-cyclodextrins (Sabadini et al., 2006; Nilsson et al., 2008).

**Property**	**α-CD**	**β-CD**	**γ-CD**
N° glucose unities	6	7	8
MW (g mol^−1^)	972	1,135	1,297
Solubility in water (g L^−1^)	145	18.5	232
Outer diameter cavity (wide end) (Å)	13.7	15.3	16.9
Inner diameter cavity (wide end) (Å)	5.7	7.8	9.5
Volume cavity (Å^3^)	174	262	427
No. water molecules^*^ inside cavity	5.8	8.7	14.2
Heat capacity (Cp) of bound water molecules	59	71	70

* *Assuming a volume of a water molecule equal to 30 Å^3^*.

From the CD spatial arrangement, the cavity shows a relatively hydrophobic character while the external surface is hydrophilic. The hydrophobic cavity enables the thread of a broad range of hydrophobic guests such as the alkyl chains of surfactants (Valente and Söderman, 2014) or aromatic compounds (Fang and Bhandari, 2010), forming highly stable host-guest complexes, and contributing for the improvement of guest properties, including solubility enhancement and volatility and antioxidant control (Gim et al., 2017; Wadhwa et al., 2017; Aytac et al., 2018).

These properties, complemented with their non-toxicity to humans make these compounds very attractive for a wide range of industrial applications (Del Valle, 2004) involving, for example, food packaging (Astray et al., 2009; Sagiri et al., 2016), pharmaceutical technology (Loftsson and Brewster, 2010; Popielec and Loftsson, 2017), and catalytic processes (Li et al., 2012; Madhulika et al., 2016; Sebastien et al., 2016).

Native CDs are, naturally, interesting hosts. They can be, however, modified in order to change the respective symmetry, or introduce specific groups in an effort to expand the respective range of applications. Modifications in CDs are, however, relatively difficult because of the similarity between the hydroxyl groups in which these modifications can be introduced. These have been carried out resorting to specific substitutions in the wide and narrow rims. A variety of derivatives has been obtained and, for example, CD derivatives of pharmaceutical interest include the hydroxypropyl derivatives of β- and γ-CDs, the randomly methylated β-CD, sulfobutylether β-CD, and also branched CDs (Loftsson et al., 2005).

### Cellulose

Cellulose is one of the most versatile polymers in the world, being considered a promising feedstock for different industries, including paper, textile, and pharmaceutical. This polymer is produced by some microorganisms and can be found primarily in plants, the main source for commercially and research activities.

Cellulose was described by Payen, in 1838, as a fibrous component of plants that was resistant to extraction with organic and aqueous solvents. Although the molecular formula of this compound was determined by Payen as C_6_H_5_O_10_, the polymeric structure of cellulose was only established by Staudinger, a century later (Lavoine et al., 2012; McNamara et al., 2015).

Cellulose is a linear homopolymer whose structural unit is cellobiose, formed by two units of anhydro-D-glucopyranose (AGUs) linked by β-1,4-glycosidic bonds. D-glucose units adopts a stable chair conformation, with the hydroxyl groups in the equatorial positions. This polymer is biosynthesized by the cellulose synthase enzyme, with polymerization through a condensation reaction, from glucose in the form of the substrate uridine diphosphate (UDP)-glucose. The glucose units are arranged in the ß configuration, along the chain, at the anomeric position. The term anhydroglucose units is used to describe each repeat unit in the cellulose backbone and is associated to the loss of a molecule of water. Depending on the origin, the degree of polymerization can range from a few hundred to a few thousand AGU units. The regular arrangement of the chain and the presence of three hydroxyl groups, in the AGU unit of this polymer, is responsible for exhibiting strong hydrogen bonds, which impart unique characteristics such as high crystallinity, low solubility in conventional solvents, and low reactivity (Shen, 2009; Shen et al., 2016; Shaghaleh et al., 2018).

Cellulose possesses microfibrils formed by crystalline and amorphous regions and, depending on the origin and isolation or processing conditions, can assume different crystal lattice structures, reason why it is considered a polymorph. Native cellulose is denoted as cellulose I and possesses a crystallinity degree of 45–60%. When treated with a concentrated solution of NaOH or precipitated, it forms cellulose II (or regenerated cellulose), a thermodynamically more stable polymorph. Besides these two polymorphs there are two others, cellulose III and IV, which can be obtained from treatment of I and II with liquid ammonia or ethylene diamine (cellulose II) and treatment of the regenerated cellulose fibers in glycerol at high temperature (cellulose IV) (Lavoine et al., 2012; Wüstenberg, 2015).

Cellulose has been used as raw material in several areas such as construction, textile and paper industry, among many others. Cellophane and rayon production paved the way for the development of new cellulose-based polymers through chemical modification. Various derivatives have been synthesized over the years, which find applications in areas as diverse as the food industry, paint production, pharmaceutical applications, personal care products, among others (Kamide, 2005).

Common approaches for cellulose modification encompass chemical, physical and biological methods. The main chemical reactions of cellulose modification can be divided into two major groups: those involving hydroxyl groups and those requiring degradation of the main chain. The former includes reactions of esterification, etherification, substitution, sulfation, phosphorylation, oxidation, and others. Among the degradation reactions, the more important ones involve the hydrolysis and oxidation of the glycosidic bonds.

The principal cellulose derivative prepared by sulfation is cellulose sulfate and agents such as SO_3_, chlorosulfonic acid and SO_3_.DMF complex can be used for this purpose. Phosphorylation can be accomplished using for example phosphoric acid or anhydride. Sodium cellulose phosphate, used in medicine to prevent the formation of calcium containing kidney stones, is synthesized via a phosphorylation reaction.

Cellulose ethers can be prepared using the Williamson synthesis. First the polymer is treated with an alkali, followed by reaction with alkyl halides. Derivatives such as methyl cellulose, ethyl cellulose, hydroxyethyl cellulose are prepared in this manner. Sodium carboxymethyl cellulose is synthesized using a similar strategy: cellulose is dissolved in a basic solution and then reacted with chloroacetic acid.

Cellulose esters such as cellulose acetate, butyrate, propionate, phthalate and acetate-butyrate are usually prepared by reaction of cellulose with the respective organic acids or anhydrides.

Degradation reactions with acids and alkalis promote the cleavage of glycosidic bonds and consequently the formation of low molecular weight polymers.

The physical modification of cellulose consists in the fragmentation of the cellulose backbone to obtain lower molecular weights. Methods such as ultrasound, microwave and gamma-ray irradiation can be used for this purpose.

Cellulose biological modification methods involve the use of enzymes or bacteria to induce degradation in the polymer chain (Koschella et al., 2006; Jain et al., 2013; Shokri and Adibkia, 2013; Li et al., 2016a).

The functionalization of cellulose and cellulose derivatives by grafting with CDs has contributed for the development of new materials with improved properties and added value, in different areas, involving the development of new materials. These include the removal of pollutants (Nthunya et al., 2017), such as metal ions (Wan et al., 2004; Zhou et al., 2011) dyes (Ogawa et al., 2011; Xiong and Shao, 2011; Ghemati and Aliouche, 2014; Song et al., 2017), polycyclic aromatic compounds (Liu et al., 2015b; Zhang et al., 2015; Yuan et al., 2017a), and drugs (Celebioglu et al., 2014; Orelma et al., 2018) from wastewaters, and enantiomeric separation (Xiao and Chung, 2007; Xiao et al., 2007; Zhou et al., 2009; Zhang et al., 2012; Yuan et al., 2017b). Also contemplated, are membrane processes (Andrade et al., 2015; Sulaiman et al., 2017) and food packaging. In the latter, particular attention has been directed to the development of active films aiming at improving the shelf-life time of perishable food. Different approaches were explored for this purpose, including (i) the grafting of CDs to cellulose nanocrystals and cellulose nano- and micro-fibers (e.g., essential oils-containing release packaging films (Lavoine et al., 2014a; Saini et al., 2016; de Castro et al., 2018; Muratore et al., 2018), and membrane processes (Andrade et al., 2015; Sulaiman et al., 2017), and (ii) the incorporation of CDs:essential oil host-guest compounds in cellulose-derivative films [e.g., cellulose acetate (Yang et al., 2017) or cellulose sulfate (Chen, 2016)]. These approaches have allowed improving the encapsulation of essential oils and thus enhancing the antibacterial, antimicrobial, and antifungal properties of bioactive packaging films.

As previously indicated, cellulose possesses unique advantages and interesting properties, such as the respective availability, mechanical robustness, stability, hydrophilicity, biocompatibility, and biodegradability (Medronho et al., 2013). However, cellulose-based materials are also stimuli-responsive, altering the behaviors in response to environmental stimuli, such as temperature and pH (e.g., environmental changes prompt cellulose-based hydrogels to display swelling or deswelling processes) (Liu et al., 2016; Iohara et al., 2017). The type of use is determined by the degree of crystallinity, conditioned by the respective origin. The major production in nature is associated to the generation of the cell walls in woody plants, however, cellulose is also produced from the seed hairs of the cotton plant, in a relatively pure form. In addition to crystallinity, the difference between different forms of cellulose is related to the shape and size of their particles. For instance, microcrystalline cellulose grades are multifunctional pharmaceutical excipients, extensively used in pharmaceutical industries, as compressibility enhancers, binders in wet and dry granulation processes, thickeners and viscosity builders in liquid dosage forms and free-flowing agents in solid dosage forms (Mogosanu and Grumezescu, 2015; Thakur, 2015; Jawaid and Mohammad, 2017).

A substantial effort of research(see e.g., Medronho et al., 2013) has allowed improving and enhancing the physical and chemical features of cellulose. Cellulose graft copolymers can be prepared by polymerization methods initiated by enzymes, chemicals, high-energy radiation, and UV rays, and characterized using mechanical analysis methods, TGA, XRD, XPS, FTIR, elemental analysis, NMR, SEM, TEM, and AFM. These derivatives have been used successfully in different applications, including food, paper, textiles, biotechnology, environment, and pharmaceutics. These result from the hydrophilic/hydrophobic character, elasticity, water absorption capacity, adhesive features, adsorption or ion exchange skills, resistance to microbial attacks, and thermal, optical, and mechanical resistance.

In the following section important contributions using CD-cellulose based materials are summarized including those related to pharmaceutical and textile technologies and a new promising field related to sensing devices.

## Application of cellulose and cyclodextrin based materials

### Pharmaceutical technology and drug delivery

In the last few years, the utilization of cellulose derivatives has seen a rapid increase for different pharmaceutical applications. One of the major reasons for this increase lies in the need for achieving critical and desired tunable properties such as biocompatibility, reduced or no toxicity, bioavailability and biodegradability.

Pharmaceutical excipients derived from cellulose and CDs have thus been extensively used (see e.g., Zhang et al., 2013; Muankaew et al., 2017) due to their “green” and specific binding properties, suitable for controlled and sustained drug delivery systems. This requires modifying the morphology and structure of cellulose for attaining the desired properties. In contrast to pure cellulose, which possesses some limitations, the most relevant being poor solubility, bad plasticity and stability, and absence of antibacterial activity, cellulose derivatives have been explored as potential materials for developing several dosage forms and drug delivery systems, aiming at achieving better therapeutic outcomes. In these systems, the gelling and solubility behavior of drugs can be modified, providing different mechanisms for controlling the respective release profiles. In this section, recent advances on drug delivery applications of cellulose-based systems are presented, covering some preparation and functionalization methods, as well as addressing relevant characteristics of the drug product, including the release behavior.

Current research on the application of cellulose for drug delivery encompasses the formulation of nanoparticles (Murali Mohan et al., 2012), microparticles (Li et al., 2016b), tablets (Jawaid and Mohammad, 2017), hydrogels (Zhang et al., 2013; Ghorpade et al., 2016; Liu et al., 2016; Gafitanu et al., 2017; Sun et al., 2017b), and transdermal drug delivery systems (Jawaid and Mohammad, 2017).

For instance, ether and ester derivatives of cellulose are widely used in the formulation of pharmacare products. These polymers display different physicochemical and mechanical properties, which are crucial in different types of pharmaceuticals such as bioadhesives and mucoadhesives, osmotic drug delivery systems, extended and controlled release matrices, and extended and delayed release coated dosage forms. Other possible applications include, compressibility enhancers (compression tablets), stabilizers and thickening agents (liquid dosage forms), binders (granules and tablets), gelling agents (semisolid preparations), among many others (Sun et al., 2017a,b).

The cellulose ethers are high molecular weight polymers obtained from replacing the hydrogen atoms of the hydroxyl groups in the anhydroglucose units of cellulose with alkyl derivatives. These are widely used, often in combination with other polymers, in various types of bioadhesives, such as ocular, nasal, buccal, and transdermal formulations. Nonionic cellulose ethers such as MC, EC, HEC, HPC, HPMC, CMC and anionic ether derivatives, such as NaCMC are examples of commonly used cellulose ethers (see Jawaid and Mohammad, 2017 and references therein). These are recognized by FDA as safe (GRAS) and are indispensable in drug delivery and pharmaceutical technology, being the basis for several drug products. The respective molecular weights, chemical structures, distribution of substituents and degree of substitution determine the commercial value of these derivatives, which present relevant properties such as solubility, viscosity in solution, surface activity, thermoplastic film characteristics and stability against biodegradation, thermal variations, hydrolysis, and oxidation. In general, cellulose esters are insoluble in water and possess good film forming characteristics, being broadly used, in its organic form, in pharmaceutical controlled release experiments, such as osmotic and enteric coated drug delivery systems. CA, CAP, CAB, CAT, and HPMCP are some of cellulose esters that have been employed in pharmaceutical research and in commercial products (Heinämäki et al., 1994). The most common available formulations possessing these polymers in the respective composition are enteric coated dosage forms. (Liu and Williams, 2002; Lecomte et al., 2003). The inorganic cellulose esters, including cellulose nitrate or pyroxylin and cellulose sulfate are less important in pharmaceutical formulations and industries, due to some inherent shortcomings, including low solubility in pharmaceutical solvents, and high flammability.

Polymers based on inclusion complexes and composed of multiple CD molecules threaded on the chain have attracted great attention in recent years for developing supramolecular materials (Guo et al., 2013; Klaewklod et al., 2015). Additionally, cellulose derivatives are optimal guest polymers for the design of novel CD-containing supramolecular complexes. The host-guest chemistry of CD/cellulose derivatives in aqueous solutions has been widely explored, especially, in drug delivery systems. For instance MC has been compared with CDs in terms of hydrophobicity, displaying affinity to the cavity of β-CD and forming amphiphilic supramolecular polymers (Du et al., 2012; Klaewklod et al., 2015).

**Figure 2:**
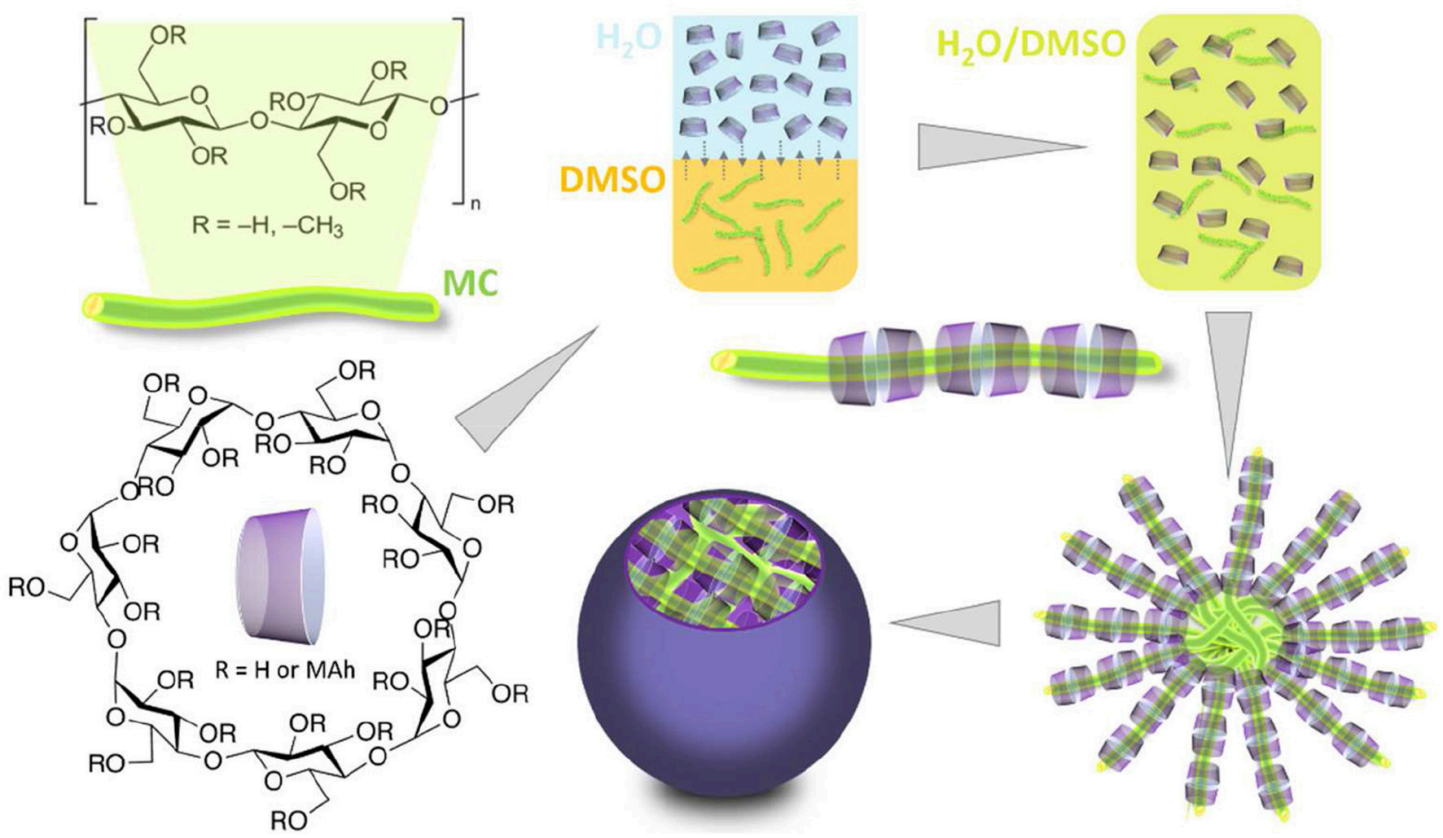
Schematic illustration of the formation of supramolecular micelles from MC, β-CD and MAh/β-CD. Adapted from Du et al. (2012).

Du et al. (2012) have constructed supramolecular polymer micelles based on maleic anhydride (MAh) modified cyclodextrin (MAh/β-CD) and MC, using a one-pot self-assembly procedure (Figure 2). The micelles displayed regular spherical shapes (with 25 ± 5 nm diameters), possessing a MC-based core and a hydrophilic shell based on β-CD and MAh/β-CD (critical micelle concentrations of 15.13 and 20.89 mgL^−1^ for MC/β-CD and MAh/β-CD:MC, respectively). The *in vitro* drug release behaviors of the micelles were established using prednisone acetate as a model drug. It was concluded that the MAh/β-CD:MC micelles possessed drug-enrichment cores and exceptional sustaining release time (ca. 700 h).

In a more recent study, Guo et al. (2013) have described a simple and ecological route for the synthesis of CD carbamates and production of cellulose-containing supramolecular polymer micelles. The respective drug release behavior was assessed using prednisone acetate, as a model drug. It was shown that the micelles derived from CD carbamate/cellulose were suitable for long time drug release. The β-CD:MC micelles displayed an accumulated release of ca. 100% within 200 h, while in the other micelles, based on γ-CD and ethyl cellulose or cellulose acetate, the sustained release of the drug was 500 h (Guo et al., 2013).

In previous years the need of suitable nanocarriers for delivering antitumor agents was the basis of sustained release studies of drug-loaded cellulose-*g***-**poly(l-lactide) micelles, formed by the copolymer, in aqueous media (Dong et al., 2008). In another study (Yan et al., 2009), strongly dependent cellulose-based dual graft molecular brushes, composed of ethyl cellulose-g-poly (*N,N*-dimethylaminoethyl methacrylate)-g-poly(ε-caprolactone) (EC-g-PDMAEMA-PCL), have revealed their potential in drug nanocarriers. Also, the delivery of antitumor doxorubicin has been potentiated by 2-hydroxyethyl cellulose-*g*-methoxy poly(ethylene glycol)-poly(ε-caprolactone) (Hsieh et al., 2008; Chen et al., 2011). The HEC-*g*-*m*PEG-PCL porous membrane, loading a drug content of 1.5 mg cm^−2^, was used as a penetration matrix for transermal delivery of catechins. This membrane enhanced the permeation up to 0.84 mg mL^−1^, in contrast to the HEC membrane which presented a limited permeation of catechin (0.04 mg mL^−1^).

The release of drugs depends on several aspects such as diffusion, swelling, and degradation/erosion of the grafted matrix and can be controlled by different environment stimuli. For instance, variations in the pH have revealed the ability of EC-*g*-PDEAEMA copolymers in the rifampicin release, i.e., the release of the drug at pH 6.6, in the buffer solution, was higher than the release at pH 7.4 (Wang et al., 2011). Different release behavior of antibiotics (e.g., cephalexin) in different media have also been observed for CMC modified via grafting of poly(hydroxyethyl acrylate) or polyacrylamide (Moghaddam et al., 2014).

Cappello et al. (2002) have developed topical formulations of rufloxacin resorting to HPMC and CDs. The latter were used to improve the solubility and ocular bioavailability of the drug. HP-β-CD formed the most soluble inclusion complex with rufloxacin. Specifically, the addition of 0.25% HPMC to the solutions containing HP-β-CD increased the solubilizing effect of CD, reducing the amount of CDs necessary for solubilization of the drug. The most promising formulation was then composed by 0.3% (w/v) of rufloxacin, 6.4% (w/v) of HP-β-CD and 0.25% (w/v) of HPMC (Cappello et al., 2002).

Host-guest complexes between modified cellulose nanocrystals (CNCs) and β-CD has been used for surface coating purposes (Castro et al., 2016; Jimenez et al., 2017) and for preparing supramolecular hydrogels, with polymers, such as Pluronic. Lin and Dufresne (Lin and Dufresne, 2013) have used doxorubicin hydrochloride as a model drug for characterizing the release behavior in these gels. Interestingly, a special drug release mechanism was observed upon fitting the release curves into the Ritger–Peppas equation. In fact, drug release followed a Fickian diffusion, when using a neat Pluronic/α-CD:doxorubicin hydrogel system. However, an anomalous transport release mechanism was observed for the *in situ* CNCs/CD-Pluronic-doxorubicin hydrogels (Lin and Dufresne, 2013; Jawaid and Mohammad, 2017).

In another study (Malik et al., 2017) different proportions of β-CD, CMC, acrylic acid and *N'N'*-methylene bis-acrylamide were used for the preparation of hydrogels via free radical polymerization, directed at controlling the delivery of antiviral drugs (e.g., acyclovir). The successful grafting of the components into the polymeric and thermodynamically stable network was confirmed using FTIR and a thermal/morphological characterization. The obtained hydrogel allowed a controlled release of acyclovir. However, a maximum of 96.15% of cumulative drug release was attained at pH 7.4, from the hydrogel containing the highest acrylic acid concentration, optimum β-CD and CMC and low *N'N'*-methylenebis-acrylamide concentrations, while exhibiting a pH dependent swelling profile (Malik et al., 2017).

Ndong Ntoutoume et al. (2016) have synthesized curcumin–CD/CNC complexes by functionalizing CNC with cationic β-CD. This was carried out by ionic association of CNC and CD and encapsulation of curcumin into the CD/CNC complexes. It was shown that these complexes displayed an antiproliferative effect on prostatic and colo-rectal cancer cell lines.

Mucoadhesive nasal gels of oxybutynin chloride have also been prepared using many different mucoadhesive polymers, including HPC carbopol and MC. *In vivo in situ* models have been used for evaluationg β-CD, EDTA and bile salts as potential nasal drug adsorption optimizers (El-Gizawy et al., 2013).

Formulations containing β-CD and chitosan based matrices and EC have been prepared for the release of ibuprofen. The effect of digestive enzymes on the physicochemical properties of those formulations was studied aiming at assessing the protective role of polysaccharides in gastrointestinal media. The most stable carrier systems were obtained using formulations composed of equal amounts of EC and β-CD, allowing a slow release of the drug until 24 h (maximum drug release of 101.04 ± 0.65 %, in which 83.08 ± 0.89 % of ibuprofen was released in colonic medium) (Rehman et al., 2013).

**Figure 3:**
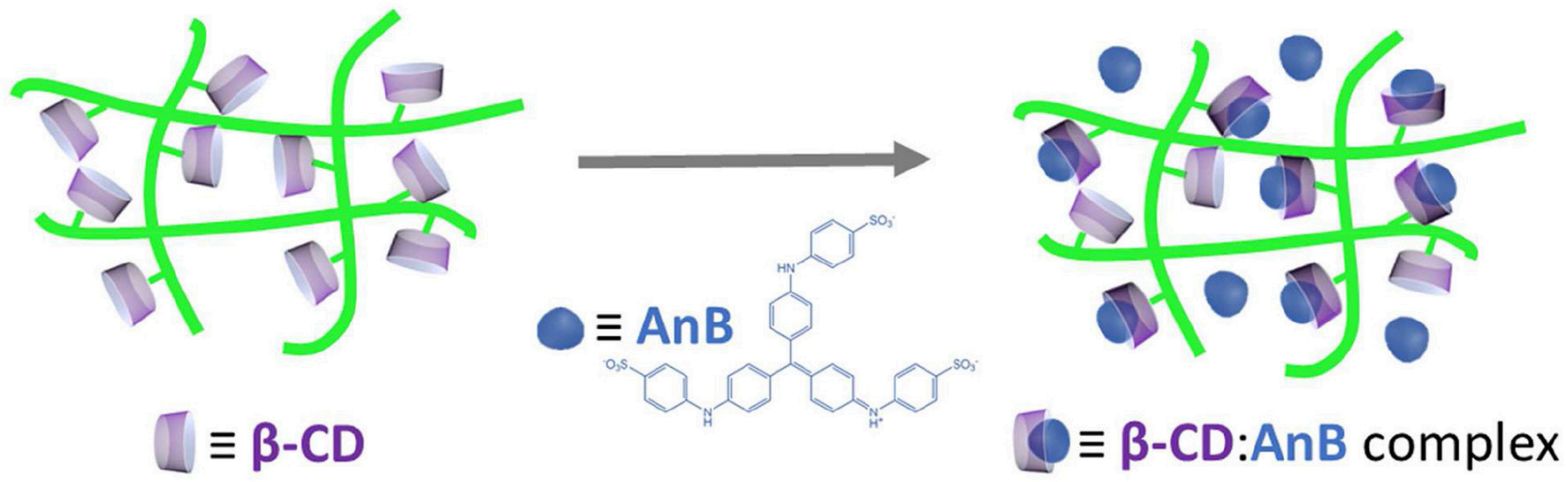
Schematic representation of the mechanism for the adsorption of AnB in the hydrogels.

β-CD/cellulose hydrogels have also been prepared by Zhang et al. (2013), in a basic solution (NaOH/urea), using epichlorohydrin as crosslinker and in the presence of 5-fluorouracil (5-FU), bovine serum albumin (BSA) and aniline blue (AnB) (see Figure 3).

It was found that an increase of the β-CD content led to a decrease of the swelling degree and the water uptake of the hydrogels. Different drug release behaviors were observed for 5-FU and BSA. Weak interactions were observed between BSA and β-CD, while the formation of β-CD:5-FU complexes inhibited the controlled release. AnB was adsorbed by the hydrogels leading to a fluorescence enhancement, which was explained by the formation of β-CD:AnB complexes. These results also confirmed the usefulness of this type of hydrogels as pharmaceutical excipients for hydrophobic drugs (Zhang et al., 2013).

Recently, Marcos et al. (2016) have developed supramolecular gels based on poloxamer, HEC and α-CD for improving the solubilization and sustained release of griseofulvin, an antifungal drug for topical application. The authors evaluated the effects of the α-CD concentration (from 0 to 10%w/w), on the phase behavior of mixtures of Pluronic P123 (14%w/w) and HEC (2%w/w), at different temperatures. Both components, poly(ethylene oxide) (PEO) blocks of Pluronic and HEC, were able to form supramolecular complexes with CD, avoiding the phase separation and improving the solubilization of the drug. The rheological and bioadhesive properties of the gels, in the presence and absence of the drug, were tuned using different proportions of the polymers. Furthermore, the addition of HEC to form P123/α-CD gels, enhanced the viscosity at 25°C and enabled optimizing the release of griseofulvin at 37°C. In addition to these features, these ternary supramolecular gels displayed favored biocompatibility and activity against *T. mentagrophytes* and *T. rubrum*, being thus considered a suitable platform for the topical treatment of dermatophytosis and also to be incorporated in formulations for long-term sustained release of several drugs.

Han and co-workers (Han et al., 2017) have obtained a hydrogel with three-dimensional double network structures, in which electrostatic and host-guest interactions play the leading roles. HEC and a modified chitosan (HACC) were crosslinked using one-pot reaction for preparing the spherical cage. HACC provided a positive charge core to attract sulfobutylether-β-CD (SEB-β-CD), the negative host molecule. The method of weight increment and photometric titration was implemented to determine the amount of SEB-β-CD loaded (ca. 50.3%). It was shown that the hydrophobic guest substance in the SEB-β-CD@hydrogel was released in a sustained manner in buffer solution and the controlled release of SEB-β-CD by ion-exchange method provided new insights on the rapid release of hydrophobic guest substances. (Han et al., 2017).

A zero-order release kinetics of glipizide, used as model drug, from compression-coated tablets for oral administration, was observed by Huang et al. (2013), using HPC as the coating layer. Those tablets were able to form a hydrogel, when in contact with the gastrointestinal fluid, which decreased the drug release rate. It was found that such release rate was controlled by the erosion of the gel matrix. Similarly to previous observations, the formation of host-guest complexes allowed enhancing the solubility of the drug in different pH conditions, thus increasing the dissolution rate of the compression-coated dosage form. It was concluded that different properties, such as the viscosity of HPC and the distribution of the drug between the outer layer and the core of the matrix, can be used for controlling the release behavior (Huang et al., 2013).

The use of β-CD derivatives proved a powerful resource to increase the solubility of chemotherapeutic agents, such as benznidazole. HPMC has been the basis for guiding the release of such agents from solid CD-based host-guest complexes, ensuring the drug bioavailability. In turn, HP-β-CD produced a significant improvement in drug solubility being often selected for the development of solid systems (Sá-Barreto et al., 2013). New solid dosage forms with improved physicochemical properties and oral bioavailability have been developed, containing HP-β-CD and hydrophilic additives, obtained from supercritical antisolvent processes. HP-β-CD NPs loading dutasteride formed aggregates within the nanoscale (a mean particle size of ca. 160 nm and a specific surface area over 100 m^2^/g). It was shown that the type of hydrophilic additive influence the maximum achieved supersaturation and also the drug precipitation rate. A maximum solubility and extended supersaturation was observed for the drug-loaded HP-β-CD nanostructures with HPC. The bioavailability of these nanoparticles was comparable to that of the commercially available formulation (Kim, 2013).

The inclusion of ciprofloxacin by grafting β-CD on cellulose fibers has been reported by Dong et al. (2014b). The β-CD-grafted cellulose was prepared by the formation of citric acid-β-CD, which was obtained via dehydration of the two adjacent carboxyl groups of the citric acid to form a cyclic anhydride which reacts with the hydroxyl groups of the β-CD portals. The grafted ratio of β-CD onto cellulose fibers was 9.7%, under [citric acid-β-CD] = 300 gL^−1^, pH 3.4, 15 min, and 160°C conditions. The release behavior of the encapsulated ciprofloxacin hydrochloride from cellulose fibers was also investigated. The CD-grafted cellulose showed a higher cumulative release (ca. 90% in half-hour) of the biocide, when compared with the native fibers. The same level was attained with the modified fibers after 240 min, as a consequence of the formation of host-guest complexes. However, the presence of β-CD and biocide contributed for an increase in the disorder of the cellulose microstructure, affecting the respective mechanical properties.

Comparing to pure fibers, the bacterial activity against *E. coli* and *S. aureus* of the grafted chains, loading ciprofloxacin, was significantly higher (Dong et al., 2014b).

**Figure 4:**
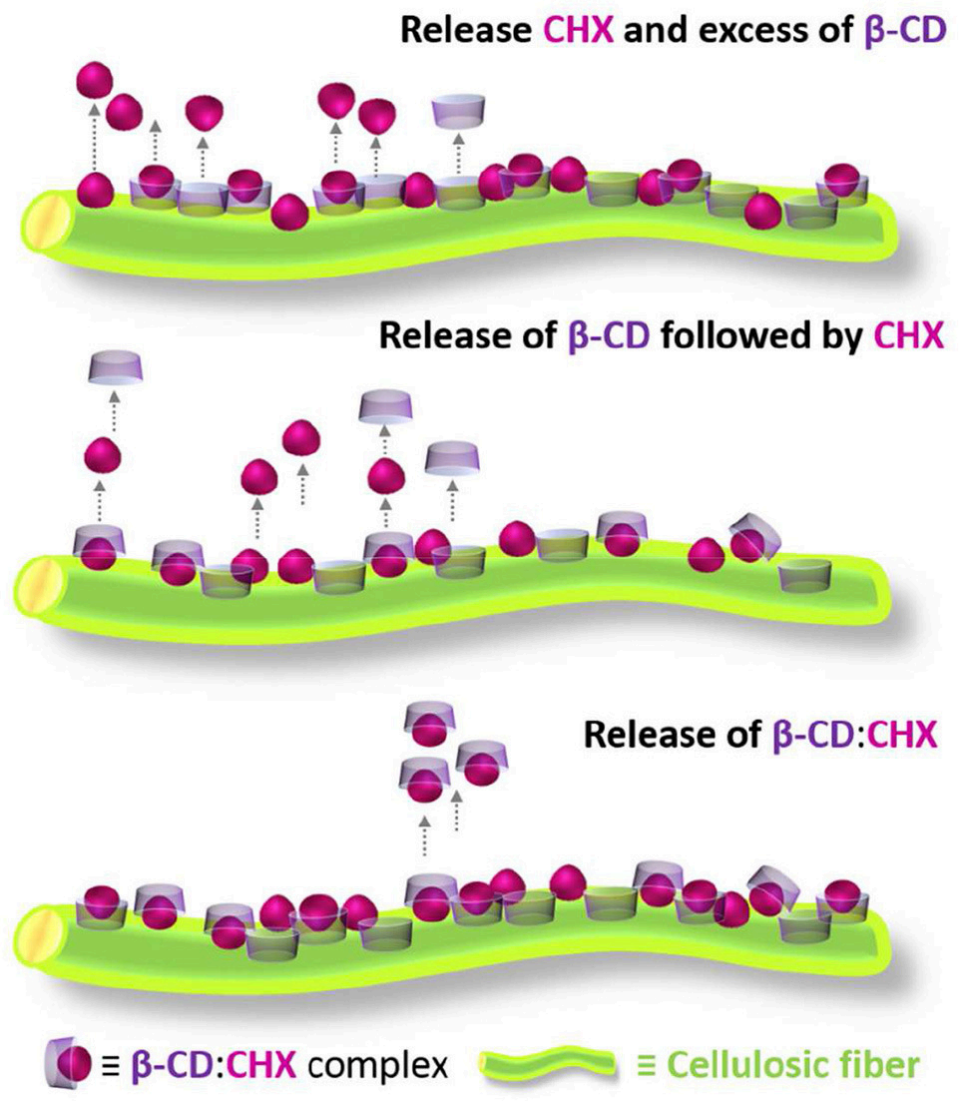
Schematic representation of the different scenarios for explaining the release mechanism involving β-CDs and chlorhexidine digluconate (CHX), and the respective inclusion complexes. Adapted from Lavoine et al. (2014b).

Lavoine et al. (2014b) have developed a high-performance delivery system using β-CD containing microfibrillated cellulose (MFC)-coated papers. A model antibacterial drug, chlorhexidine digluconate (CHX) was used in a suspension of MFC, a β-CD solution, or mixed with both MFC and β-CD, before coating onto a cellulosic substrate (see Figure 4). The proposed scenarios for explaining the release mechanism suggested that (i) the β-CD:CHX complexes are linked to cellulose via β-CD/cellulose interaction, being associated to a longer release of the drug, (ii) the interaction with cellulose can be done through the CHX moiety protruding from the β-CD macrocycles, which are firstly released, followed by the CHX molecules, (iii) either CHX or β-CD, in the supramolecular form, are concomitantly linked to cellulose. In the latter scenario, the complexes are released at a low kinetic rate, as a consequence of the chemical interactions between the β-CD:CHX and cellulose.

Comparing the β-CD and MFC releases of CHX, the former promotes a continuous and longer release profile, due to the formation of the inclusion complex with CHX. Furthermore, when the two compounds were combined a complementary action was observed. The βCD:CHX-coated sample released the lowest amounts of CHX for more 10 h than the CHX/MFC-coated sample. MFC promotes the burst effect, while β-CD controls the amount of drug released over time. It was concluded that the combination of β-CD and MFC is suitable for the rapid delivery of CHX, promoting the sustained release of active compounds (Lavoine et al., 2014b).

Other approaches have been tested for prolonging the drug release in aqueous solutions. These include recent works on the use of electrospun fibers composed by HPC and complexes of HP-β-CD-sulfisoxazole. These components were encapsulated in poly(ε-caprolactone) nanofibers which allowed a more sustained drug release (Aytac et al., 2015; Costoya et al., 2017).

### Cellulose-CD interactions for textile applications

The use of CDs and their derivatives in the textile domain has attracted many researchers and industry (Buschmann, 2001; Szejtli, 2003; Shown and Murthy, 2009; Voncina and Vivod, 2013) due to the possibility to develop new functional materials with added value. Due to the ability of CD to form inclusion complexes with a wide range of guest compounds, including those with antimicrobial (Voncina and Majcen, 2004), insecticide (Szejtli, 2003; Medronho et al., 2013), and fragrance (Singh et al., 2017) properties, several strategies and approaches have been developed to bind CD to cellulose or cellulose-based fabrics.

The first report on the grafting of CDs onto cellulose fibers was published in 1980 by Szejtli et al. (1980), corresponding to a condensation reaction, using epichlorohydrin as a crosslinking agent. The incorporation of CD by chemical and physical routes has been started in the end of the last century (Poulakis et al., 1992; Denter and Schollmeyer, 1996; Denter et al., 1997b).

**Scheme 1:**
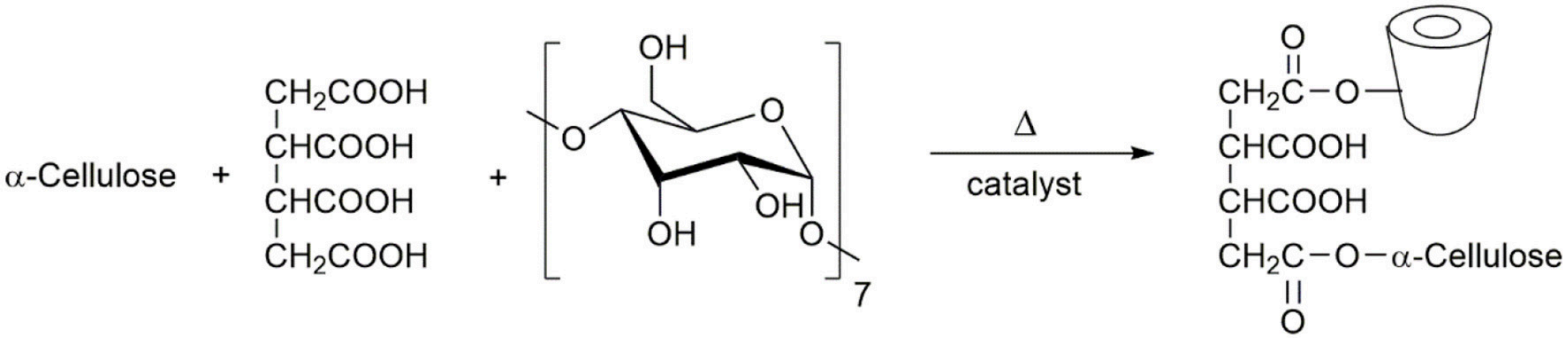
The esterification reaction between cellulose and β-CD in the presence of polycarboxylic tetra acid.

A well-known approach for chemical modification of cellulose through cyclodextrins is by using polyacids. Polyacids, such as the 1,2,3,4-butane tetracarboxylic acid (BTCA), can act as anchors, by the esterification of hydroxyl groups of both cellulose and cyclodextrin (Morris et al., 1995; Martel et al., 2002; Voncina and Marechal, 2005; Bhaskara et al., 2014). This strategy has the advantage of using acids with different degrees of deprotonation, depending on a previous tretament with sodium hydroxide. The grafting of α-CD to cellulose via BTCA (Scheme 1) occurs at high temperatures (>170°C) both in the presence and in the absence of catalyst (e.g., sodium hypophosphite), and the esterification yield is, in general, evaluated by weight gain (Voncina and Marechal, 2005). The grafting process is effective with a weight gain higher than 30% by using 8% of β-CD. In the case of using cotton fabric the grafting yield is 17% by using ca. 20% of β-CD. The modification will improve the wrinkle recovery angle, whitness index and wettability; on the other hand, the tensile strength decreases around 17% (compared with unttreated fabric) (Abdel-Halim et al., 2014). In the case studies involving α-cellulose (Medronho et al., 2013) it has been found that both BTCA and CD can act as cellulose crosslinker affecting drastically the cellulose solubility.

**Scheme 2:**
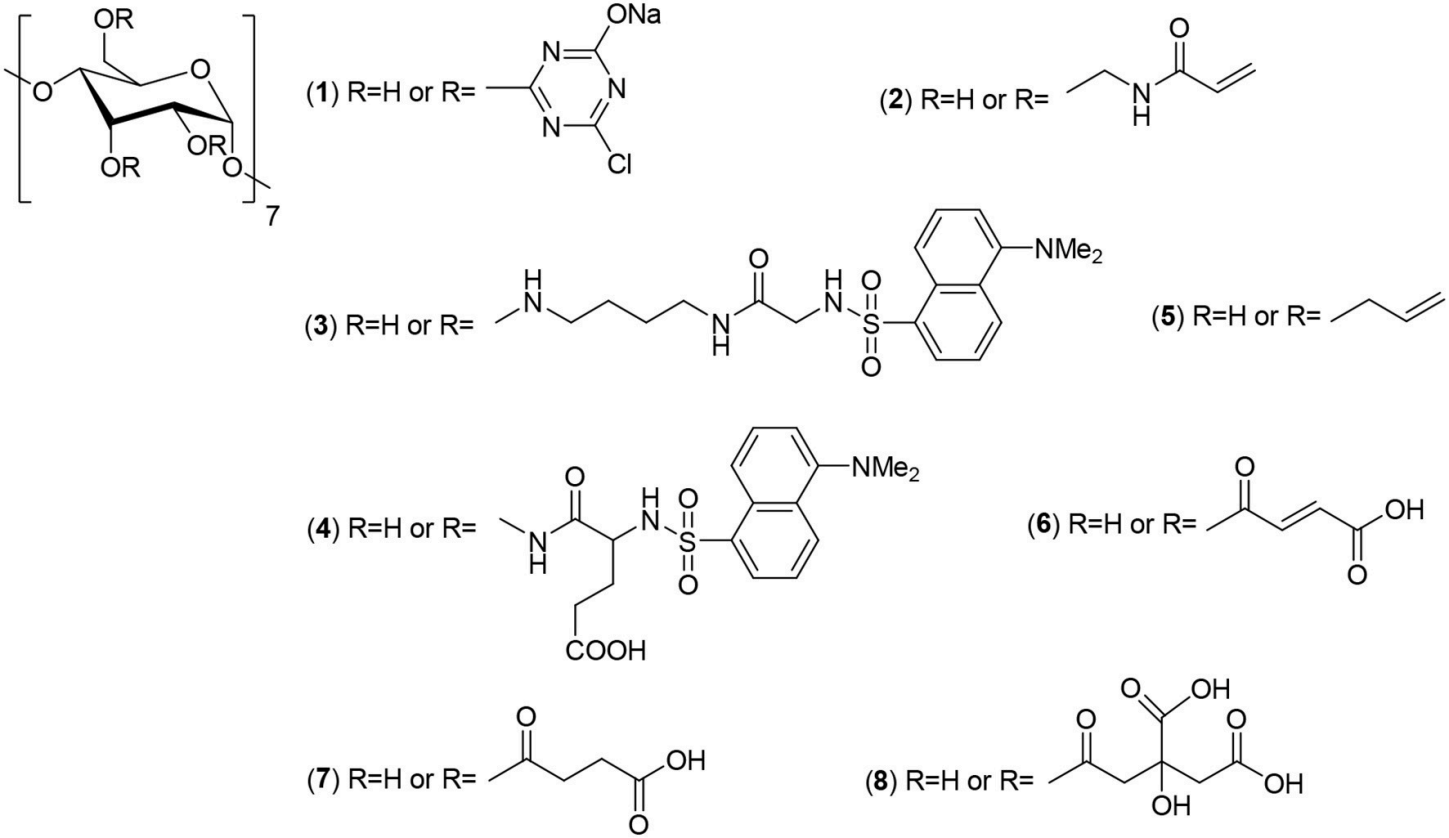
CD-derivatives used for cellulose functionalization, including monochlorotriazinyl-β-CD (1), acrylamidomethyl-β-CD (2), dansyl-based compounds [ansylglicine (3) and dansyil L-glutamate (4)], allylcyclodextrin (5) and β-CD modified with fumaric (6) or succinic (7) acids. Also included is citric acid used for anchoring β-CD, via host-guest interactions.

Diacids can also be used for the functionalization of cellulose surface materials, such as CNCs—the crystalline domains extracted from cellulosic materials by acid hydrolysis (Tang et al., 2017). Although the impregnation of CNC by β-CD can lead to the incorporation of the latter in a percentage of 0.8%, the use of β-CD modified with fumaric (6) or succinic (7) acids (Scheme 2) in solutions increase the content of CD onto CNC surface to 6.1 and 5.6%, respectively. The functionalization was then used for the incorporation and consequent controlled release of carvacrol-a major constituent of oregano essential oil and an efficient antimicrobial agent (Arrieta et al., 2014); however, it was found that the amount of carvacrol loaded into functionalized CNC was only 34% higher than the corresponding native CNC (Castro et al., 2016).

Several CD-derivatives have also been used for cellulose functionalization (see Scheme 2). Monochlorotriazinyl-β-CD (MCT-CD) (1) has been prepared by modification of cotton cellulose surface (Hebeish and El-Hilw, 2001) allowing, for example, an improvement the wrinkle proofing capacity and the preservation of wettability (Popescu and Sandu, 2014; Popescu et al., 2014). The process is inspired in a well-known dyeing process (Denter et al., 1997a), which consists in the reaction of an amino-substituted dye with 2,4,6-trichloro-1,3,5-triazine to obtain a chlorotriazine-functionalized dye which couples with nucleophilic surface groups (Zollinger, 2003). The binding of MCT-CD to the cellulose surface allows the additional treatment with dyes and/or protective resins. However, the functionalization extent is quite dependent on the CD concentration and a higher dosage of CD is required. It should be highlighted that an advantage of this chlorotriazine chemistry is the use of water as solvent.

The dyeing process can also be carried out using a different methodology, in which a host-guest compound is initially formed between the dye and a cyclodextrin and, consequently, the supramolecular complex is bound to the cotton surface (Craig et al., 2001). This will improve the stability of the dye and, consequently, increases the life time of the material. A strategy to reach that goal is to initially form highly stable rotaxane complexes by using a diazo dye and hexakis (2,3,6-tri-*O*-methyl)-α-cyclodextrin, followed by the covalent binding of the of the chlorotriazine part of dye to the cellulose hydroxyls (Craig et al., 2001).

The grafting of MCT-β-CD onto microcrystalline cellulose was also reported (Rehmann et al., 2003) by using two different simple approaches (differing in the amount of water and in the presence/absence of NaCl) yielding in a CD substitution degree of ca. 3 and 20%, respectively. The final product was then tested for the inclusion of limonene, a multipurpose compound used as a flavor modifying agent. The functionalization of cotton fabric to obtain flame retardant properties has also been reported (Veerappagounder et al., 2016). For that purpose, the cotton fabric was modifed with natural CDs and MCT-β-CD, and using BTCA as crosslinker (see Scheme 1). Diammonium phosphate was tested as the flame retardant. This compound confers retardant properties, but it also contributes to the aging of cellulose-based materials. It was observed, however, that the tretament of the MCT-CD-cotton with diammonium phsophate not only enhances the flame retardation but also the durability of the fabric, when compared to the unmodified cotton (Veerappagounder et al., 2016).

The grafting of cellulose cotton fibers with acrylamidomethyl-β-CD (2) (Scheme 2) leads to a functionalization yield, in optimal conditions (ceric ammonium nitrate, *T* = 60°C, 1 h), of 65%, occurring essentially at the cotton surface (Lee et al., 2001). This process occurs initially, by the derivatization of CD with *N*-methylolacrylamide (NMA), at acidic medium; the formation of a carbonium ion of the NMA is then reacting with the hydroxyl groups of CD. This functionalization provides one more vinyl group in the modified CD, which is used to react with hydroxyl groups of cellulose.

The oxidizing agent of cellulose is a key factor for the grafting of MCT-β-CD; the effect of Ce(IV), able to generate reactive sites on the cellulosic backbone, has been tested by Lo Nostro et al. (2003) to modify the surface of fabric composed by cellulosic fiber extracted from wood pulp (so-called tencel) by grafting with acrylamidomethylated-β-CD and MCT-β-CD. The tencel has been used because it is similar to cotton but possesses a higher crystallinity (Crawshaw and Cameron, 2000) and, consequently, shows a higher breaking load, higher resistance to laundry cycles, etc. (Woodings, 1995). The aroma and antimicrobial activity has been checked by using vanilline, and benzoic acid and iodine, respectively, as guest agents. The latter showed to be effective against *S. aureus* and the fragrance lifetime of the modified tencel fabric has increased two times, when compared with the untreated fabric.

As described above, the large majority of functionalization routes involves the previous modification of CDs. It is know that the primary hydroxyl groups of cyclodextrins can be oxidized to the corresponding aldehydes and carboxyl groups by 2,2,6,6-tetramethylpiperidine-1-oxyl (TEMPO), in the presence of sodium hypochlorite (Delattre et al., 2009) or alternatively in the presence of laccase (Marzorati et al., 2005). Yu et al. (2016) have demonstrated that β-CD can be oxidized and grafted onto wool fibers, by using a one-pot Schift base reaction. The reaction involves the modification of CD hydroxyl groups to aldehydes, in the presence of TEMPO/laccase followed by reaction with amino groups of the wool.

The use of corona discharge surface has been an alternative for the chemical modification of cellulose. This is a solvent-free process and only affects the cellulose surface without changing the bulk properties (Lei et al., 2000; Benhadi et al., 2011). Two different grafting methodologies were tested: in the first one the cotton was initially impregnated with CD (an allyl-cyclodextrin, (5) (Scheme 2) and then was subject to corona discharge; in the second one, the surface of cotton was initially modified and, afterwards, immersed in a CD solution. The weight gain obtained was similar in both cases. The CDs kept their ability to form host-guest compounds; however, the mechanism of CD-cellulose interaction was not unveiled (Benhadi et al., 2011).

A relevant problem associated with the cellulose modification is the assessment of the degree of substitution and, which is of outmost relevance, the degree of availability of the CDs to form host-guest inclusion compounds. The latter is probably more relevant taking into account that CDs can make part of other reactions involving, for example, polymer crosslinking (Cho and Workman, 2015). Based on the high stability of host-guest compounds formed by alkyl- and cyclo-amines and cyclodextrins (Valente and Söderman, 2014), a method based on the interaction of different types of amines (primary, secondary, and tertiary ones) has been developed (Grechin et al., 2007). From all amines tested, the most suitable one for the aforementioned purposed objective is cyclohexylamine, not only because forms a stable host-guest interaction compound but also because it does not interact with the textile surface avoiding an over quantification of grafted the CD.

The use of ink-jet printing technology in fabrics and other textile materials is highly spread throughout the textile industry. However, ink-jet technology still has some drawbacks when compared to traditional technologies (Zhao et al., 2017). There are aqueous-dye (Elgammal et al., 2016) and pigment-based inks (Cie, 2015), both types presenting their own advantages or disadvantages. However, in terms of sustainability and green concerns emphasis must be put into water-based pigments. Taking advantage of the ease with which CD can form host-guest compounds with hydrophobic molecules, the incorporation of CD into the process has been considered. For example, aiming at improving the ink-jet printing performance in cotton, β-CD was introduced, by using citric acid (8) as anchor and sodium hypophosphite as catalyst, by using a dip-pad-dry-cure process. Among some of the evaluated properties, the color fastness is one of the most relevant ones, once it is related with the stability/aging of the ink-fiber system. The cotton fabrics modified with CD display both a washing and crocking fastness significantly higher than the corresponding unmodified fabric, indicating that the presence of CD contributes for a better fixation of the ink, probably due to the formation of a dye-CD supramolecular compound (Zhao et al., 2017).

The development of biofunctional textiles for transdermal delivery has attracted the attention of biomedical community and textile industry (Labay et al., 2014; Seib et al., 2014). For this purpose, several finishing methods have been reported (Salaün et al., 2012; Chatterjee et al., 2014); among them, the use of CDs is seen as a means to improve the release properties of drugs. The use of a β-CD nanosponge for functionalization of cotton fibers has been reported by Mihailiasa et al. (Mihailiasa et al., 2016). Cyclodextrin nanosponge are insoluble, 3D hyper-crosslinked polymers. Different crosslinks can be used: 1,1′-carbonyldimiidazole (CDI), diphenyl carbonate, or organic dianhydrides (Caldera et al., 2017). By using an excess of 1,1′-carbonyldimiidazole (1:8, CD:CDI mol/mol), at 80°C, the dehydrated CD can form solid monolith nanosponges which are, after that, incorporated into cotton. The efficiency of the modification has been assessed by using phenolphthalein as a probe for the presence of CD (Kuwabara et al., 1998; Chen et al., 2017). For studying the topical and delivery properties of the modified fabric, melatonin has been used as model drug.

**Figure 5:**
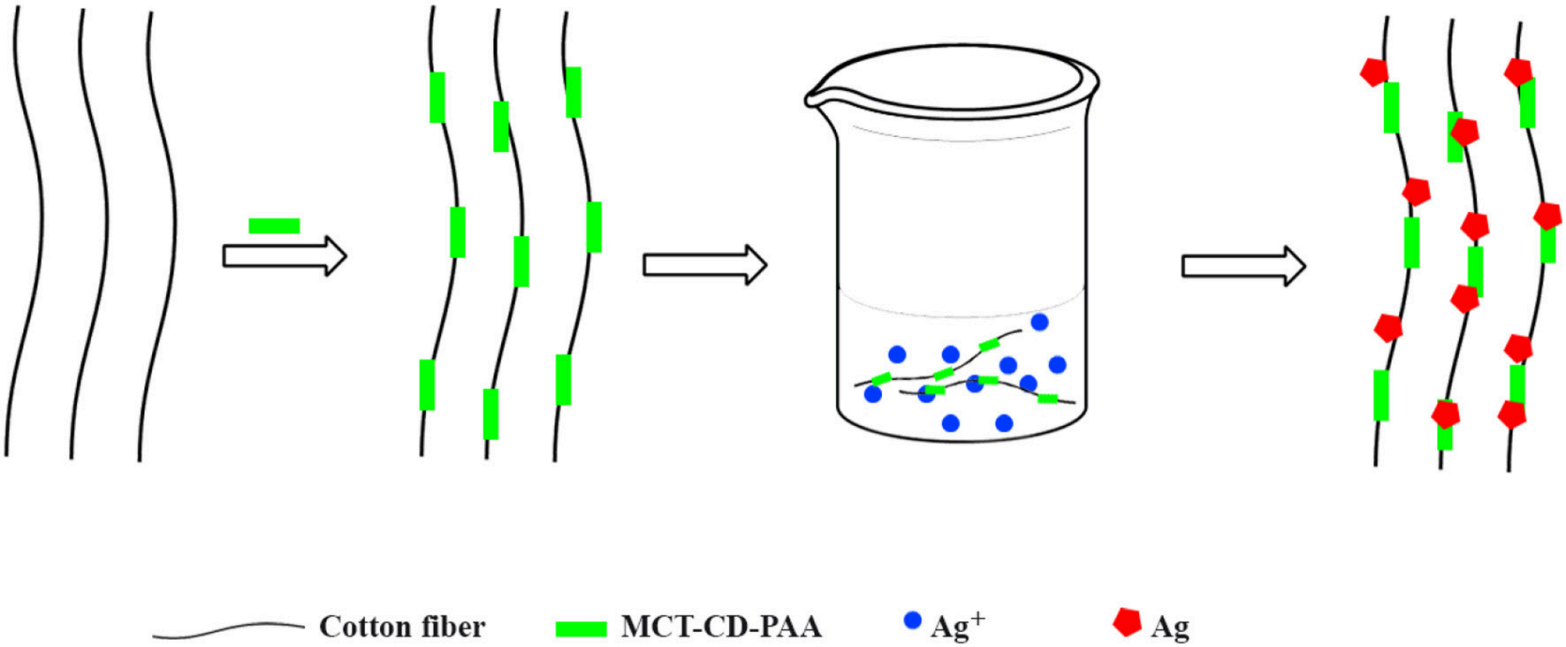
Schematic representation of the grafting of cotton fibers followed by the loading of Ag NPs.

The manufacture of antibacterial textiles is determined by the want for hygienic and clean goods. These can be produced resorting to various methods, without compromising the quality of the fabrics. Silver is a popular antimicrobial agent which displays high cytotoxicity toward a broad range of microorganisms (Pollini et al., 2009). There are different strategies for the application of Ag into cellulose-based fabrics, some of which will be mentioned. Hebeish et al. (2014b) have reported the incorporation of silver nanoparticles (NPs) in cotton grafted with CD derivatives. In particular, they have modified CDs by reacting either MCT-β-CD or native β-CD with poly(acrylic acid) (PAA) with subsequent reaction with the cotton. A slightly modified route involves the previous cationization of cellulose with 3-chloro-2-hydroxypropyl trimethyl ammonium chloride, at basic pH, followed by interaction with MCT-β-CD-PAA or β-CD-PAA (Hebeish et al., 2014a). The incorporation of Ag NPs is made by sorption of silver nitrate in the modified cotton, followed by reduction of Ag(I) to Ag(0) by using β-CD-*g*-PAA or NaBH_4_, respectively (Figure 5). It was concluded that in the case of β-CD-*g*-PAA-containing cotton the wet and dry crease-recovery have improved when compared with native cotton fabric; concomitantly, the modified cotton showed an effective antimicrobial action against *Escherichia coli* (*E. coli*) and *Staphylococcus aureus* (*S. aureus*) either by using PAA or NaBH_4_ as silver reducing agent. On the other hand this method has shown a drawback related with the change of the fabric color to brown as a consequence of the formation of Ag NPs (Hebeish et al., 2014b).

Based on the same principle other antimicrobial agents have been tested (Peila et al., 2013; Dong et al., 2014a,b). For example, β-CD has been grafted to cotton fabric by using BTCA as cross-linker (Medronho et al., 2013) and further loaded with octenidine dihydrochloride –an antimicrobial agent; the modified cotton fabric shows antimicrobial action against two bacteria (*E. coli* and *S. aureus*) and two fungi (*Candida albicans* and *Aspergillus flavus*), whilst without CD no antibacterial agent is sorbed into the cotton.

Considering the advantages of the inclusion of TiO_2_ NPs in fabrics, one of which consists in reducing the photo-yellowing of wool (Montazer and Pakdel, 2010) and nano-photo scouring of cotton (Montazer and Morshedi, 2012), the development of functionalized cotton fabrics with antimicrobial and self-cleaning properties have been achieved by using composite complexes of Ag/TiO_2_ with CDs (Attarchi et al., 2013). The study was carried out by incorporating the composite into cellulosic cotton by using three different procedures: *in-situ*, pad-dry-cure, and exhaustion (Attarchi et al., 2013). In the first method, the interaction between the composite and cellulose can be physical adsorption, hydrogen-bonding or ionic; in the case of the exhaustion procedure, the interaction is merely physical and in the latter case, the post-treatment is done in the presence of citric acid, ensuring the covalent bonding to cellulose. The exhausting method has proved to be the more efficient in the synthesis of antimicrobial and self-cleaning efficient cotton fabric; besides, it was demonstrated that the incorporation of CD complexes does not affect the fabric properties (Attarchi et al., 2013).

### Sensors

Chemosensors based on CDs and immobilized onto cellulose have several advantages, including being easily disposed of. There are several examples of these type of sensors. Tanabe et al. have developed modified-β-CD fluorescencent probes used dansyl-based compounds (Ikunaga et al., 1999): dansylglicine (3) (Tanabe et al., 2001b) and dansyil L-glutamate (4) (Tanabe et al., 2001a). These probes are based on the fact that the substituted dansyl group (Scheme 2) is able to form intramolecular host-guest compounds. In the presence of analytes, there will be a competition between the “new” guest molecule and dansyl group, which affects the fluorescence of the probe. The immobilization of β-CD-derivatives onto cellulose membranes was carried out by the initial oxidation of cellulose using sodium periodate, followed by condensation through the 1,6-hexanediamine group of the oxidized cellulose and the modified cyclodextrin. The obtained sensors were tested toward a set of cholic acid derivatives, showing a selectivity directly dependent on the binding constant between β-CD and the guest molecules.

**Figure 6:**
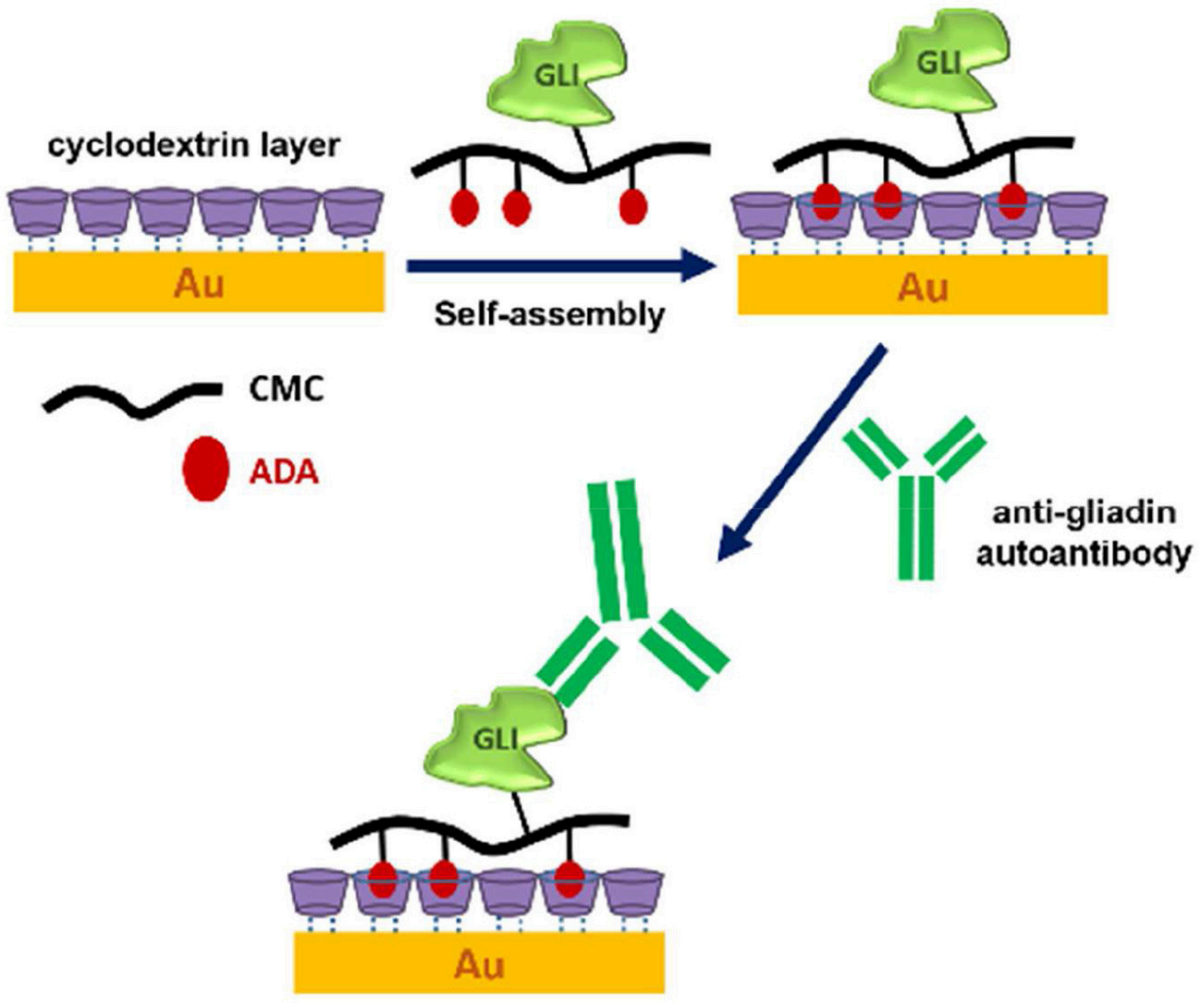
Schematic representation of a possible approach for auto-antibody detection. The modification of the gold surface (Au) with CD allows the preferential interaction with adamantine (ADA)-appended antigen carriers (GLI-CMC-ADA). Reproduced from Ortiz et al. (2011) (with permission of the Royal Society of Chemistry).

Other approaches (Ortiz et al., 2011) have explored the interaction of CD-modified surfaces (e.g., CD with Au-nanoparticles) and adamantane-appended antigen carriers (see Figure 6). Considering the higher selectivity of the CD cavity toward adamantine, the latter is grafted to CMC allowing the formation of a layer on the modified Au-NPs. In turn, the CMC backbone is used as an antigen carrier, and the obtained layer-by-layer-based probe is able to detect electrochemically the presence of anti-gladin antibodies. The quantification of antibodies has been comparable to that of ELISAtests, with a deviation of around 10% (Ortiz et al., 2011).

## Concluding remarks

The design of advanced materials based on cellulose and cyclodextrin derivatives is still a challenging and ingenious proving-ground, from both the academic and commercial points of view. Several approaches are currently introduced to produce cellulose derivatives with improved properties that can be applied in different scenarios. However, the broad range of properties and specificities for different target molecules, such as drugs, ensures that one specific type of polymer will not always be suitable of effectively interacting and releasing with such molecules, thus requiring new polymer candidates. Comprehensive rationales of the involved interaction mechanisms from computation are still much needed. This means that substantial efforts involving chemical-physics, synthetic organic chemistry, must be combined with work on materials design and characterization, and drug delivery for obtaining products that make a change.

## Author contributions

TC, AP, and AV conceived the review. AP and AV reviewed the MS for scientific/technical merit. TC, DM, AP, and AV shared in writing the article.

### Conflict of interest statement

The authors declare that the research was conducted in the absence of any commercial or financial relationships that could be construed as a potential conflict of interest.

## Footnotes

^1^Reuters T. Web of Knowledge [Online]. *Available:* *http://apps.webofknowledge.com* [*Accessed January* 2018].

## Abbreviations

5-FU: 5-fluorouracil
ADA: adamantine
AGU: anhydro-D-glucopyranose
BSA: bovine serum albumin
BTCA: 1,2,3,4-butane tetracarboxylic acid
CA: cellulose acetate
CAB: cellulose acetate butyrate
CAP: cellulose acetate phthalate
CAT: cellulose acetate trimellitate
CD: cyclodextrin
CDI: 1,1′-carbonyldimiidazole
CHX: chlorhexidine digluconate
CMC: carboxymethyl cellulose
CNC: cellulose nanocrystal
EC: ethyl cellulose
EDTA: ethylenediaminetetraacetic acid
ELISA: enzyme-linked immunosorbent assay
FDA: Food and Drug Administration
GLI: gliadin
GRAS: generally recognized as safe
HEC: hydroxyethyl cellulose
HPC: hydroxypropyl cellulose
HPMC: hydroxypropylmethyl cellulose
HPMCP: hydroxypropylmethyl cellulose phthalate
MAh: maleic anhydride
MC: methyl cellulose
MCT: Monochlorotriazinyl
MFC: microfibrillated cellulose
NMA: N-methylolacrylamide
NP: nanoparticles
PAA: poly(acrylic acid)
PCL: poly(ε-caprolactone)
PDEAEMA: poly[2-(diethylamino)ethyl methacrylate]
PEG: poly(ethylene glycol)
NaCMC: sodium carboxymethyl cellulose
SEB: sulfobutylether
TEMPO: 2,2,6,6-tetramethylpiperidine-1-oxyl
UDP: uridine diphosphate.
